# Cyranose^®^ 320 eNose Effectively Differentiates Pre- and Post-Challenge Respiratory Samples in an Induced Bovine Respiratory Disease Model

**DOI:** 10.3390/vetsci12060578

**Published:** 2025-06-12

**Authors:** Conrad S. Schelkopf, Leslie F. Weaver, Michael D. Apley, Roman M. Pogranichniy, Lance W. Noll, Jianfa Bai, Raghavendra G. Amachawadi, Brian V. Lubbers

**Affiliations:** 1 Department of Diagnostic Medicine/Pathobiology, College of Veterinary Medicine, Kansas State University, Manhattan, KS 66506, USA; conrads@vet.k-state.edu (C.S.S.); rmp1@vet.k-state.edu (R.M.P.); lwnoll@vet.k-state.edu (L.W.N.); jbai@vet.k-state.edu (J.B.); 2 Department of Clinical Sciences, College of Veterinary Medicine, Kansas State University, Manhattan, KS 66506, USA; lfweaver@vet.k-state.edu (L.F.W.); mapley@vet.k-state.edu (M.D.A.); agraghav@vet.k-state.edu (R.G.A.)

**Keywords:** bovine respiratory disease, cattle, diagnostic test, electronic nose, volatile organic compounds

## Abstract

Field-based diagnostic technologies which aid in the early detection of bovine respiratory disease (BRD) are of great need, given the rising attention related to animal welfare and antimicrobial stewardship. This induced BRD study followed 12 Holstein calves through pre-challenge (day 1–3) and post-challenge (day 6–13) periods with daily sampling of nasal secretions with nasal swabs and expired air with air collection bags for determination of BRD status by use of an electronic nose (eNose). Animals were challenged with bovine herpes virus-1 (BHV-1) on day 3 following sample collection and *Mannheimia haemolytica* on day 5. Results demonstrated a high degree of accuracy for the eNose in correctly classifying pre-challenge samples for nasal swabs (93.5%) and expired air (96.8%). Post-challenge correct classification by the eNose was 97.8% for nasal swabs and 72.5% for expired air samples. Logistical regression was used to determine the probability of agreement between eNose classification and actual animal BRD status by study day. The largest discrepancy between nasal swab and expired air samples fell on days 6 and 7, immediately following the bacterial challenge. The eNose demonstrated potential as a field-based diagnostic tool for the detection of BRD with nasal swabs as the optimal sample type.

## Simple Summary

Diagnostic tools that can accurately detect pneumonia in cattle on-farm and provide early, consistent, and easy-to-interpret results are sparse. This study tested a handheld electronic nose (eNose) to determine its capability in accurately diagnosing cattle experimentally infected with a common virus and bacteria of cattle pneumonia. Additionally, multiple sampling methods were tested to determine the optimum sample type for use on the eNose. When samples collected from animals are tested on the eNose, the device provides a single result related to the pneumonia status of the animal, making implementation on-farm straightforward for the device operator. Results showed that the eNose was able to accurately classify animals based on their pneumonia status. Nasal swab samples were the ideal sample type for use on the eNose due to better accuracy and ease of use. The eNose’s ability to provide accurate and easy-to-interpret results related to pneumonia status have the potential to promote better antimicrobial stewardship and animal welfare through consistent and early detection of this disease.

## 1. Introduction

Bovine respiratory disease (BRD) is the most significant health challenge facing United States commercial cattle feeding operations and consequently results in substantial economic losses for the industry each year [1]. Costs commonly associated with BRD include prevention, treatment of sick cattle, decreased productivity in the form of lower average daily gains, death loss, and labor. The development of field-based diagnostics for the early detection of BRD is gaining increasing attention, driven by growing concerns over animal welfare and the rising threat of antibiotic resistance in the livestock industry [2]. The ability to provide rapid, accurate, and easy-to-interpret results, which can be implemented shortly after diagnosis to guide timely treatment, has shown to be a definitive need for the cattle industry. However, validation of field-based diagnostic tools is complicated by the lack of an affordable and practical gold-standard diagnostic test [3].

Currently, antemortem diagnosis of BRD in commercial cattle feeding operations is primarily accomplished by animal caretakers based on visual observation of clinical signs. Clinical illness scoring systems, such as the Wisconsin [4] and California scoring systems [5] for pre-weaned dairy calves and the less structured DART (depression, appetite loss, respiratory character, and temperature) system [6] for feedlot cattle, have been used to provide more objective criteria for BRD diagnosis and treatment. Unfortunately, these systems rely on proper training of the animal caretaker to determine a final diagnosis with test accuracy largely based on the individual scorer’s ability to detect clinical signs [7,8]. Due to a consistent lack of accuracy in diagnosing BRD from clinical signs alone, ancillary tests, such as body temperature measurement [9], thoracic auscultation and lung ultrasound with associated scoring systems [10,11,12,13], are occasionally implemented to help in the diagnosis. Regrettably, these tools and associated scoring systems also require training of the animal caretaker before implementation and still vary widely based on the animal caretaker’s skill in operating such modalities [14].

Field-based diagnostic tests which provide objective results pertaining to a BRD-specific diagnosis and depend only minimally on the skill of an individual in operating and accurately interpreting the test are limited. Computer-aided lung auscultation (CALA) is one of the only validated field-based technologies for feedlot cattle in the diagnosis of BRD [15]. CALA allows diagnostic results to be consistent from test operator to operator [16]. Although not validated or widely used in a field setting, an on-farm colorimetric detection assay utilizing loop-mediated isothermal amplification (LAMP) has been tested for the detection of common BRD bacterial pathogens, including *Pasteurella multocida*, *Mannheimia haemolytica*, and *Histophilus somni* [17]. It detects the presence of these specific BRD bacteria in respiratory samples based on a color change detected by computer software. A limitation inherent to diagnosing BRD with LAMP pertains to its sensitivity in detecting BRD pathogens which are commensals of the bovine respiratory tract.

Another field-based diagnostic technology which is not validated or widely used in a field setting for the diagnosis of BRD is the electronic nose (eNose). This device detects volatile organic compounds (VOCs) emitted from a biological sample via an internal bank of chemical sensors [18]. VOCs found within the respiratory tract are products of viral, bacterial, and inflammatory origin [19,20,21]. eNoses analyze sample VOC profiles and output categorical results based on pre-training of the device for specific disease processes or pathogens, thus allowing the animal caretaker to implement the device on-farm with ease. An eNose has been previously used to test nasal secretions obtained via nasal swabs of calves naturally infected with BRD (*Mycoplasma bovis* and bovine adenovirus-3). The eNose was able to correctly classify 10 calves as either “sick” or “healthy” with 100% accuracy [22]. Other studies have utilized an eNose for the detection of bovine tuberculosis in cattle. Cho et al. [23] demonstrated differentiation of 11 bovine tuberculosis-infected serum samples from 10 bovine tuberculosis-free serum samples using principal component analysis. Additionally, Fend et al. [24] were able to correctly classify 16 unknown sera samples from calves as either infected or uninfected with *Mycobacterium bovis* using a discriminant function analysis model. A single study assessed the ability of an eNose to characterize blood serum samples from cattle experimentally infected with *Mannheimia haemolytica* and non-infected cattle [25]. Results of this study compared eNose sensor responses to acute-phase protein concentrations with differentiation between infected and uninfected animals. The existing literature is deficient in studies demonstrating the use of a commercially available eNose for detecting BRD due to pathogens commonly found in United States cattle feeding operations.

This study utilizes the commercially available Cyranose^®^ 320 eNose in calves experimentally induced with BRD pathogens (bovine herpes virus-1 [BHV-1] and *Mannheimia haemolytica*) to determine its capability for accurate BRD status classification. The objectives of this study are to determine the ability of the eNose to correctly differentiate calves pre-BRD challenge from the same calves post-BRD challenge and to optimize the sample type for use on the eNose to diagnose BRD.

## 2. Materials and Methods

### 2.1. Experimental Design and Enrollment Criteria

This study was conducted over a two-week period in July and August of 2022 at the Kansas State University College of Veterinary Medicine. The study population consisted of twelve 150 kg (range: 143–172 kg) intact male Holstein calves, approximately 5 months of age, sourced from a commercial dairy. Calves were transported approximately 129 km directly from the commercial dairy to the study location where they were acclimated overnight on the day of arrival (acclimation—study day 0) before the study commenced. Calves included in the study were not previously vaccinated or treated for respiratory disease at the source location and were determined to be clinically healthy by a large-animal veterinarian on arrival to the study location. Lung ultrasound was performed on each calf prior to enrollment. No lung lesions were observed on ultrasound for any of the animals enrolled in the study. Prior to study initiation, calves (n = 12) were individually identified with a single ear tag and then were randomly assigned to two equal groups (n = 6). Two groups of six calves each were housed in separate pens with an open-front shed and 9.2 × 18.4 m concrete pad. Study calves were fed a ration containing corn, oats, and soybean meal with monensin and ad libitum access to prairie hay and water. 

During the study period, 264 respiratory samples, consisting of 132 expired air and 132 nasal swab samples, were collected from the 12 study animals. Throughout the study each calf was observed at least twice daily for signs of clinical illness. Prior to study initiation a treatment protocol was established for animals meeting clinical illness criteria in accordance with the on-site attending veterinarian. Sample collection and clinical illness observation were conducted by CSS, LFW, BVL, and MDA. On study days 1 and 2, each calf had respiratory samples collected once per day. Half of the study population (n = 6) had expired air and nasal swabs collected in the morning while the other half had respiratory samples collected in the evening. After respiratory sample collection, samples were transported to a laboratory at the Kansas State University College of Veterinary Medicine for analysis by the Cyranose^®^ 320 eNose. Calves alternated between morning and evening sample collection on each consecutive day throughout the study period except for day 3. On study day 3, samples were collected from all calves in the morning and then all animals were challenged with BHV-1 immediately after. On study day 4, calves were only observed for clinical illness with no respiratory samples collected. On the morning of study day 5, calves were challenged with *Mannheimia haemolytica* and then followed with clinical observation every six hours for a 24 h period. Twice-daily respiratory sample collection along with clinical observation resumed for all calves on study day 6 and continued through study day 13, following the same format as detailed on study day 1 and 2. The study timeline (Table 1) illustrates a simplified sampling scheme with associated viral and bacterial challenge timepoints for the live animal portion of the study. On study day 14, all calves were euthanized and necropsied for assessment of lung pathology. Calves were sedated with xylazine (IM, 0.15 mg/kg) and euthanized by captive bolt and intravenous infusion of supersaturated magnesium sulfate. Gross necropsy was conducted on all study animals, and a total percent lung consolidation was estimated by a single investigator (BVL) using the equation described by Fajt et al. [26].

### 2.2. Viral Challenge Preparation and Inoculation

BHV-1 (Colorado strain) was propagated at 37 °C in a 5% CO_2_ incubator on a bovine nasal turbinate (BT) cell line. After 70–80% cytopathic effect (CPE) development in the 75 cm^2^ cell culture flask, the material was exposed to two freeze/thaw cycles. The cell debris was centrifuged at low speed (1500× *g*) for 10 min. The supernatant was titrated in the vials and placed on the BT cells for 48 h to determine 10^5^ Tissue Culture Infectious Dose 50 (TCID50)/mL of the challenge inoculum. Individual challenge doses (4 mL) were aliquoted and stored at −80 °C. Viral challenge aliquots were thawed in a household refrigerator (~4 °C) 24 h prior to inoculation. On study day 3, all animals were restrained in a squeeze chute with a rope halter and inoculated with 4 × 10^5^ TCID50 BHV-1 by fully inserting a 5 cm plastic nasal cannula into the left nostril.

### 2.3. Bacterial Challenge Preparation and Inoculation

A field strain of *Mannheimia haemolytica* serotype A1 (genome information is available at https://www.ncbi.nlm.nih.gov/nuccore/CP017519 (accessed 11 June 2025)) was grown from a characterized clinical isolate on sheep blood agar in 5–7% CO_2_ for 18–24 h. A single isolated bacterial colony was inoculated into brain–heart infusion (BHI) broth and incubated for 16–18 h at 37 °C. The bacterial inoculum was centrifuged at 3000× *g* for 15 min at 4 °C and washed twice with phosphate-buffered saline (PBS). After the second wash, the bacterial pellet was suspended in PBS to reach an optical density equivalent to 1.0–1.2 × 10^9^ CFU/10 mL. The bacterial challenge preparation was completed the morning of inoculation (study day 5). After preparation, the inoculum was placed on ice in a light-protected cooler and transported to the study site. At the study site, all calves were restrained in a squeeze chute with a rope halter and inoculated with 10 mL of the *M. haemolytica* suspension via endoscopy into the tracheal bronchus. Following instillation of the inoculum, the endoscope was flushed with 60 mL of sterile PBS solution to achieve a total volume of 70 mL.

### 2.4. Expired Air and Nasal Swab Collection

Expired air and nasal swab samples were collected for eNose analysis. Samples were collected from all study animals once daily on study days 1, 2, and 6–13, split between two sampling timepoints (morning and evening). Six animals were sampled in the morning and six animals were sampled in the evening, with cattle alternating between morning and evening collection on each consecutive day. Only one sampling timepoint was used on the morning of study day 3 prior to the viral challenge. No respiratory samples were collected on day 4 and day 5 (*M. haemolytica* challenge day).

#### 2.4.1. Expired Air

**Figure 1:**
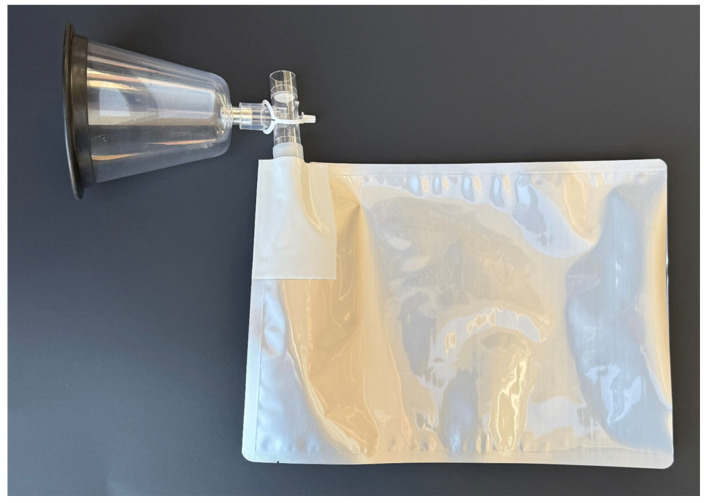
Expired air collection apparatus consisting of a non-rebreathing T-piece connected to a large canine anesthetic induction mask and a 3.79 L mylar food storage bag.

Prior to expired air collection, calves were restrained in a squeeze chute and a rope halter was applied to the calf’s head to limit movement during expired air collection. Expired air samples were collected using the apparatus depicted in Figure 1. A large canine anesthetic induction mask was connected on one end to a disposable non-rebreathing T-piece. On the outflow port of the T-piece, a sealed 3.79 L mylar food storage bag was attached via a 22 mm internal diameter (ID) tubing adapter. The anesthesia mask provided an air-tight seal around the calf’s mouth and nostrils and was held on the calf for the entire collection cycle. As the calf exhaled, expired air was diverted through the non-rebreathing T-piece into the mylar bag. As the calf inhaled, the valve on the non-rebreathing T-piece connected to the mylar bag closed and the valve opposite opened, which allowed fresh air into the mask. Calves would breathe into the air collection apparatus until the mylar bag was full (approximately 5–10 breaths). Once collection was complete, the 22 mm ID tubing adapter connected to the mylar bag was removed from the non-rebreathing T-piece and sealed with a 22 mm ID rubber tapered plug. After expired air collection, expired air bags were placed into a plastic storage container for safe transport. The bags were held at environmental temperature (~20–38 °C) after collection until transport to the laboratory where they were held at room temperature (20 °C) to be analyzed by the eNose.

#### 2.4.2. Nasal Swab

Prior to nasal swab collection, calves were restrained in a squeeze chute and a rope halter was applied to the calf’s head to limit movement during nasal swab collection. Dirt and debris were removed from the external nares with a paper towel; a 15 cm sterile rayon swab with a polystyrene handle was inserted approximately 7.5 cm into the nasal cavity and rotated across the nasal mucosa for approximately 5 s. The swab was then removed from the nares and placed in a 6 mL preservative-free blood collection tube and capped. After collection, the blood tubes containing the swabs were stored in a plastic storage container. The swab was held at environmental temperature (~20–38 °C) after collection until transport to the laboratory where it was held at room temperature (20 °C) to be analyzed by the eNose. Calf nostril sampling was alternated (left/right) each consecutive day of the study.

### 2.5. eNose Procedure

Respiratory samples were analyzed by the Cyranose 320^®^ eNose according to the manufacturer’s general recommendation and previous work performed by Schelkopf et al. [27]. The Cyranose^®^ 320 eNose is a portable, handheld device composed of 32 carbon-based sensors with potential to differentiate a wide array of VOC profiles. The Cyranose 320^®^ eNose instrument settings for analysis of expired air and nasal swab samples are outlined in Table 2 and Table 3. For expired air samples, a 16 G × 1.5” aluminum hub needle was penetrated through the mylar bag. The needle was attached to a 76 cm intravenous extension set connected to the eNose sample inlet. Prior to running the expired air samples, the eNose underwent a conditioning phase as recommended by the manufacturer, consisting of a six-minute purge cycle followed by three pre-sniffs (sample runs) using expired air samples from the previous collection timepoint. Morning and evening samples were saved and maintained in their original collection container until the next collection timepoint to be used as pre-sniff in the conditioning phase of the eNose. Pre-sniffs performed at the start of the study consisted of three additional samples collected from the study animals at the first collection timepoint. Expired air samples were analyzed between one to four hours post-collection.

**Figure 2:**
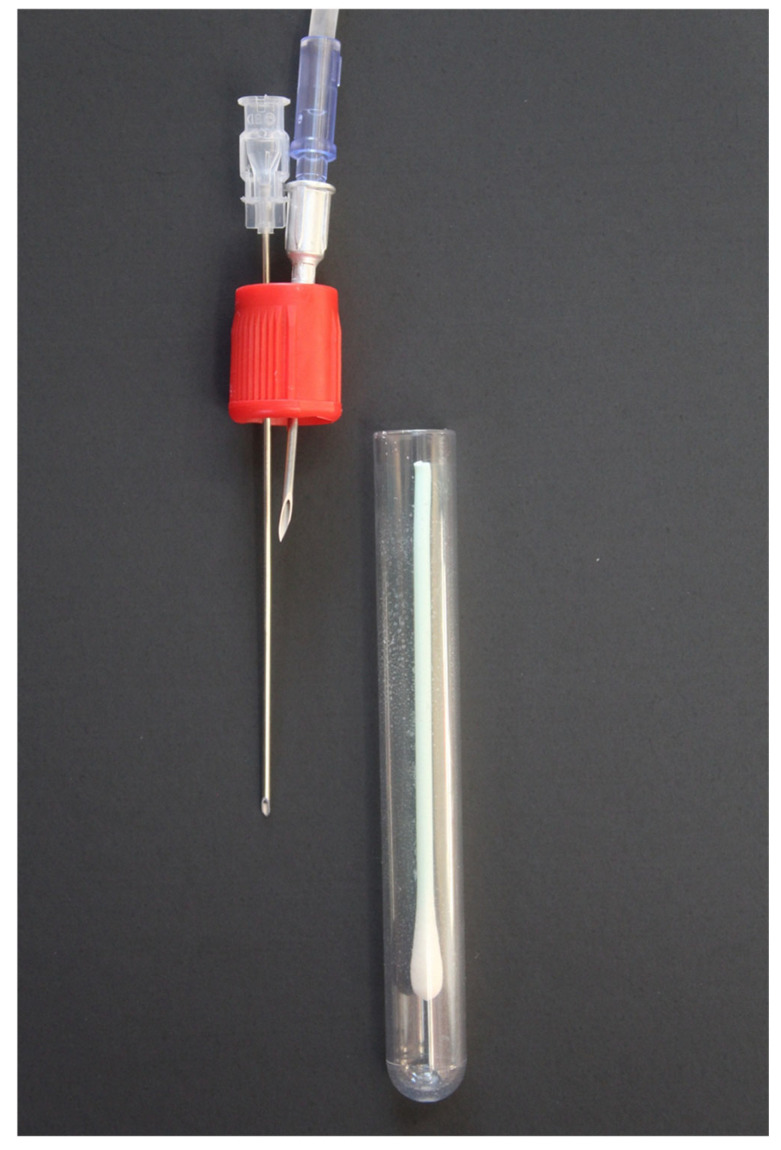
Nasal swab collection system with lid uncapped from blood collection tube storing a nasal swab. A 16 G × 1.5” aluminum hub needle attached to an IV extension set and an 18 G × 3.5” spinal needle are penetrated through the rubber cap of the tube.

For nasal swab samples, a 16 G × 1.5” aluminum hub needle was penetrated through the rubber cap of the preservative-free blood collection tube. The needle was attached to the eNose by the same set-up outlined for the expired air sample. An additional 18 G × 3.5” spinal needle was penetrated through the rubber cap of the blood collection tube next to the previous needle (Figure 2) to eliminate negative pressure generated from the eNose inlet pump. Prior to running the nasal swab samples, the eNose underwent a conditioning phase with three pre-sniffs using nasal swab samples from the previous collection timepoint as described for expired air samples. Nasal swab samples were analyzed between one to four hours post-collection.

### 2.6. eNose Training and Data Processing

All samples collected during the study were analyzed by the Cyranose^®^ 320 eNose. Raw data generated from a single sample run on the eNose was streamed into the PCnose™ software, provided with the Cyranose^®^ 320 eNose, on an external computer and stored as a comma-separated value (CSV) file. CSV files were then loaded into the Chemometric Data Analysis (CDAnalysis™) software, provided with the Cyranose^®^ 320 eNose, on the external computer to create a training set. A separate training set was created for expired air and nasal swab samples. “Post-Challenge” training set classes for both expired air and nasal swabs were created by selecting 5 sample runs on study day 13 from calves with the greatest amount of lung consolidation on necropsy (day 14). “Pre-Challenge” classes used in the training sets differed between expired air and nasal swabs and were created by selecting 5 sample runs randomly without replacement from the first 3 days of the study excluding calves used in the “Post-Challenge” class of the training set. Training sets were evaluated on the PCnose™ and CDAnalysis™ software for quality assurance and quality control, as recommended by the eNose manufacturer. The two parameters evaluated were cross validation and Mahalanobis distance, both of which provide objective measurements pertaining to how well the 2 classes in the training set are separated. Once the training sets were established, the remaining individual sample data (CSV files) from previous sample runs collected on the eNose were ran as “unknown samples” in the CDAnalysis™ software to obtain a binary outcome (“Pre-Challenge” or “Post-Challenge”). Data analysis configuration settings used for the training sets are included in Table 2 and Table 3.

### 2.7. Statistical Analysis

All expired air and nasal swab samples not used in the creation of training sets were run through their associated training set on the CDAnalysis™ software as “unknown samples” and assigned either a “Pre-Challenge” or “Post-Challenge” classification as described above. Nasal swab samples were exclusively run on the single nasal swab training set and expired air samples were exclusively run on the single expired air training set. Descriptive statistics were used to determine the count and associated percentage of correctly identified pre- and post-challenge samples on the eNose for expired air and nasal swabs.

Data were also analyzed by a logistic regression model using the “glmer” function in the ‘lme4’ package of R Studio^®^ (R Studio^®^, version 2024.9.0.375; R Core Team) to determine the probability of agreement between the eNose/CDAnalysis™ software and the animal’s actual status. The model’s outcome variable was the comparison of the classification of eNose/CDAnalysis™ (“Pre-Challenge” or “Post-Challenge”) to the actual animal status (pre-challenge vs. post-challenge). Comparison was binomial with agreement (1) or disagreement (0). The model included fixed effects for sample type, day, and the interaction of sample type by day with an associated significance level of *p* < 0.05. Animal identification was included as a random intercept in the model to account for repeated measures. The “emmeans” function in the ‘emmeans’ package of R Studio^®^ was used to calculate the probability of agreement between the eNose/CDAnalysis™ software and the actual animal status for each sample type by study day.

## 3. Results

### 3.1. Descriptive Statistics

Calf lung consolidation scores collected at necropsy (day 14) are individually reported in Table 4. Mean lung consolidation among all 12 calves was 13.17% (median: 11.85%). Cross validation of expired air training set performed on the PCnose™ and CDAnalysis™ software from the Canonical Discriminant Analysis algorithm (CDA) model was 70%, with a Mahalanobis distance between the two classes (“Pre-Challenge” and “Post-Challenge”) of 14.3. Nasal swab cross validation was 70% as well, with a Mahalanobis distance between the two classes of 5.018.

**Figure 3:**
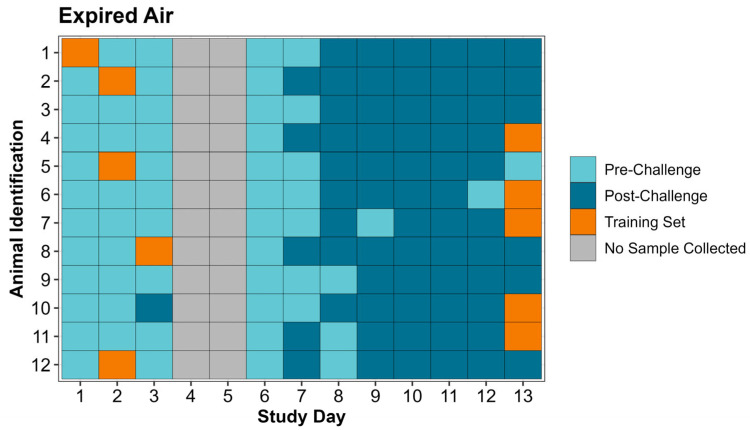
Tile map identifying individual animal expired air sample classifications by the Cyranose^®^ 320 eNose and identification of samples used in the training set by study day.

**Figure 4:**
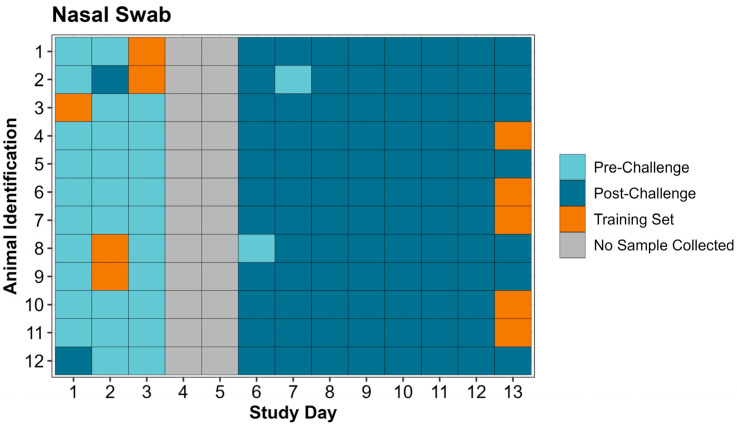
Tile map identifying individual animal nasal swab sample classifications by the Cyranose^®^ 320 eNose and identification of samples used in the training set by study day.

In total, 132 expired air and 132 nasal swab samples were collected during the study period; 10 expired air and 10 nasal swab samples were used to create the respective training sets, leaving 122 nasal swabs and 122 expired air samples for analysis by the eNose/CDAnalysis™ software. In the pre-challenge period (days 1–3), the eNose correctly identified 30/31 expired air samples as pre-challenge, compared to 29/31 nasal swab samples (Table 5). In the post-challenge period (days 6–13), the eNose correctly identified 66/91 expired air samples as post-challenge, far fewer than the 89/91 correctly identified nasal swab samples (Table 5). Individual animal eNose classification by day of the study is displayed for both expired air and nasal swabs in Figure 3 and Figure 4, along with identification of samples used in the creation of the training set.

### 3.2. Logsitic Regression

**Figure 5:**
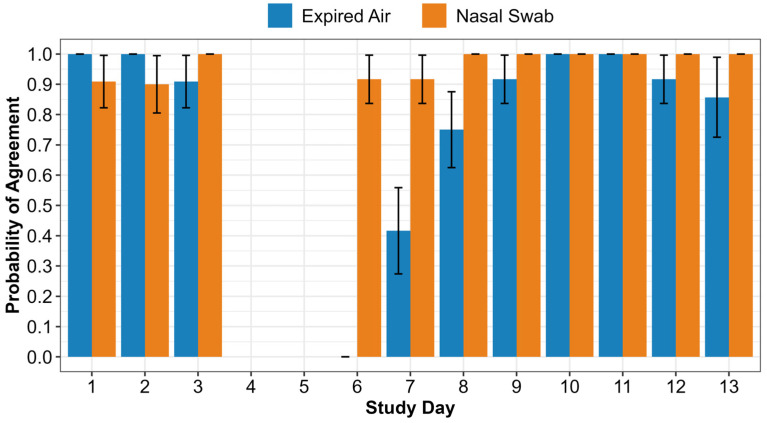
Model-estimated mean probability of agreement between Cyranose^®^ 320 eNose and actual animal status by sample type and study day. Error bars represent standard error (SE) of probability of agreement.

Figure 5 displays the model-estimated mean probability of agreement between the Cyranose^®^ 320 eNose and the actual animal status by sample type and study day. When the logistic regression model was used to predict probability of agreement as described in the methods, the model failed to converge with animal identification accounting for repeated measures. Animal identification was subsequently removed from the model. In the final model, the interaction of sample type by day was significant (*p* = 0.0493), so main effects were not reported. In the pre-challenge period, expired air had a mean probability of agreement of 1.0 (standard error [SE] < 0.01) for study day 1 and a mean probability of agreement of 1.0 (SE < 0.01) for study day 2. On study day 3, expired air had a mean probability of agreement of 0.91 (SE = 0.09). Pre-challenge results were similar for nasal swabs with a mean probability of agreement of 0.91 (SE = 0.09) on day 1, 0.90 (SE = 0.09) on day 2, and perfect agreement (probability [*p*] = 1.0, SE < 0.01) on day 3. Post-challenge mean probability of agreement differed the most between expired air and nasal swabs on study days 6 and 7 (days 1 and 2 post-*M. haemolytica* challenge [expired air—day 6: *p* < 0.01 +/− <0.01 SE, day 7: *p* = 0.42 +/− 0.14 SE; nasal swab—days 6 and 7: *p* = 0.92 +/− 0.08 SE]). On days 8 through 13, nasal swabs had perfect mean probability of agreement (*p* = 1.0 +/− <0.01 SE) between the eNose and the actual animal status. Expired air mean probability of agreement increased from day 6 to perfect agreement (*p* = 1.0 +/− <0.01 SE) by days 10 and 11. The mean probability for expired air samples decreased after day 11 to 0.92 (SE = 0.08) on day 12 and 0.86 (SE = 0.13) on day 13.

## 4. Discussion

No published study has used the Cyranose^®^ 320 eNose in the detection of BRD in cattle from respiratory samples in an induced disease model. The focus of the current study was to provide proof of concept that the Cyranose^®^ 320 eNose has potential as a BRD diagnostic tool and to assess its ease of use for potential field-based application. The methods utilized to operate the Cyranose^®^ 320 eNose were based on prior research to detect ketosis in dairy cattle by our research group and the general recommendations for basic operation provided by the manufacturer of the device [27]. Operation of the device was carried out in a manner that could be practically applied in a commercial livestock operation for the diagnosis of BRD.

In the absence of a practical and affordable gold-standard test for diagnosing naturally occurring BRD, the authors elected to use a challenge model with two commonly isolated BRD pathogens (BHV-1 and *M. haemolytica*) to test the utility of the eNose for BRD diagnosis. The challenge model provided the advantage of knowing when animals became exposed to the pathogens and allowed for daily sampling throughout the early period of known infection. Daily sampling after the BRD challenge was included in the study design to determine how early in the disease process the eNose could reliably make a diagnosis, if feasible at all. To minimize the impact of prior respiratory disease on study results, animals used in this study were selected based on no previous history of BRD treatment and the absence of BRD signs (labored breathing, nasal and/ocular discharge, cough, and lethargy) throughout the pre-challenge period. The inoculation of BHV-1 and *M. haemolytica* served as the standard method for classifying animals with BRD. The presence of BRD was confirmed on the final day of the study by evidence of lung consolidation.

Overall, the Cyranose^®^ 320 eNose was able to correctly identify pre- and post-challenge samples with a high degree of accuracy (Table 5). Specifically, in the pre-challenge period, expired air and nasal swab sample types both provided a high probability of agreement between eNose classification and actual animal status regardless of the day (Figure 5). In Figure 5, when evaluating the post-challenge period for both sample types, in general, the agreement increased as the challenge period progressed. This is not unexpected as the eNose was trained with day 13 samples, when study animals likely would have had more developed respiratory disease. The post-challenge period provided the greatest divergence between the two sample types. While both expired air and nasal swabs had the lowest probability of agreement in the post-challenge period on days 6 and 7, the nasal swab provided results that were still comparable to the high probability found in the pre-challenge and middle to late post-challenge periods.

The results in this study closely match the only other published research which used an eNose to provide a binary diagnosis for BRD [22]. Kuchmenko et al. [22] were able to correctly classify 5/5 calves as “healthy” and 5/5 calves as “sick” with an eight-sensor eNose trained on 20 healthy and 20 sick cattle from the same cohort. While it is common among studies using an eNose in the diagnosis of respiratory disease to analyze individual sensor responses [22,25], the current study uses the combined response of all 32 sensors present in the device to provide a singular categorical output. Individual sensor data were not explored in this study due to the high degree of accuracy already acquired from the combination of all sensors. However, the Cyranose^®^ 320 eNose can turn on and off any of its 32 sensors, so further optimization of the device is possible through this method. Additionally, the Cyranose^®^ 320 eNose comes equipped with other algorithms for data analysis. Only one algorithm (CDA) was applied for data analysis throughout this study due to the high degree of accuracy achieved. While the model cross validation using the CDA algorithm achieved for both training sets would be considered suboptimum (70%), previous work by Schelkopf et al. [27] determined that model cross validation on the Cyranose 320^®^ eNose did not reliably translate to diagnostic accuracy.

It is important to note that the eNose used throughout this study was trained on raw respiratory samples collected from live cattle enrolled in the study. The eNose was not specifically trained to detect certain bacteria, viruses, or specific inflammatory responses present in respiratory disease cases. Additionally, the eNose was optimized by sample selection to differentiate live animals with no clinical BRD signs from live animals which had lung consolidation on necropsy ranging from 12.30% to 24.65%. It was no surprise to the researchers that samples representing animals immediately post-BRD challenge, which likely have a minimal amount of lung consolidation, had lower agreement than those samples later in the post-challenge phase of the study. Of note, clinical signs of illness observed from the study animals did not warrant treatment of any calf at any timepoint throughout the study. Animals did not experience severe BRD signs, denoting that the eNose was trained on animals experiencing moderate to mild BRD signs only.

In terms of field application, this study differs primarily due to the collection of all samples and associated data files prior to creating a training set and running unknown sample files on the designated training set through the CDAnalysis™ software after study completion. Ideally, samples could be analyzed in real time after collection on an eNose that is already trained to detect BRD and provide results within minutes. Sample type selection in this study was also based on ease of on-farm application consisting of a hand-made expired air collection bag and a nasal swab with an associated collection system. Aside from the nasal swab providing better statistical results, it was also much easier to store, transport, and collect from cattle. The use of a blood collection tube with a pierceable rubber cap to store and analyze the nasal swab with the eNose ensures sample headspace stability. The nasal swab and blood tube system also provided better biosecurity, as collection tools were not shared between cattle, and collected samples were protected from human exposure after collection. These results and observations suggest that the nasal swab is the ideal sample in this study and warrants its use in future similar studies. While only a single nasal swab was collected from a one nostril during the sampling of each calf, no discernible difference in nasal laterality was noticed in the results of the study. In an attempt to replicate a fast-paced commercial setting, sampling one nostril rather than both provides a much more efficient collection process and limits materials needed to perform the test.

A primary limitation of this study pertains to the inability of the logistical regression model to account for repeated measures. This is likely due to the small sample size used throughout this study and the high probability of agreement within individual sampling days. Additional limitations of this study surround its applicability to naturally occurring BRD in commercial cattle feeding operations. Currently, it is undetermined how applicable the specific training set used in this study is on other cohorts of cattle or other pathogens of the BRD complex. Published studies diagnosing respiratory disease in cattle with an eNose have only trained an eNose on the same cohort it used for detection [22,25]. It is unknown if one universal training set can be created and applied to accurately detect BRD in other diverse cattle cohorts and if training sets created in challenge studies are applicable to naturally occurring disease. Sample stability after collection is also unknown. In the current study, samples were collected and then transported to a lab for analysis on the eNose in a span of one to four hours. It is undetermined if results from samples run on the eNose immediately after collection or samples run many hours to days later could still provide reliable results. While work has been carried out to characterize specific VOCs present in respiratory secretions from cattle with BRD [28,29], this was not attempted in the current study. It is unknown what exact VOCs produced by specific biological processes or organisms are used by the Cyranose^®^ 320 eNose to differentiate animals pre- and post-BRD challenge. 

## 5. Conclusions

The Cyranose^®^ 320 eNose correctly identified pre- and post-challenge respiratory samples with a high degree of accuracy. The majority of incorrect post-challenge classifications occurred immediately following BRD challenge. Given both the accuracy and logistics of sample collection, nasal swabs were the optimum sample for detection of BRD using the eNose. This study demonstrates the potential use of the Cyranose^®^ 320 eNose as an on-farm BRD diagnostic tool. Future research to confirm these findings in naturally occurring BRD cases is warranted.

## Figures and Tables

**Table 1:** Study timeline of respiratory sample collection and bovine respiratory disease (BRD) challenge by study day. “X” means that all 12 animals enrolled in the study underwent the associated procedure on the designated study day.

Procedure	Study Day												
Expired Air Collection	X	X	X			X	X	X	X	X	X	X	X
Nasal Swab Collection	X	X	X			X	X	X	X	X	X	X	X
Bovine herpes virus 1 (BHV-1) Challenge			X ^1^										
*Mannheimmia haemolytica* Challenge					X								
	1	2	3	4	5	6	7	8	9	10	11	12	13

^1^ BHV-1 challenge was administered following respiratory sample collection on study day 3.

**Table 2:** Cyranose 320^®^ eNose instrument settings for analysis of expired air samples as displayed in the PCnose™ software provided with the device.

**Class Assignment**	**Class Name**	**Training Samples**
Class 1	Pre-Challenge	5
Class 2	Post-Challenge	5
**Flow Operations**	**Time (seconds)**	**Pump Speed (cm^3^/minute)**
Baseline purge	10	High (180)
Sample draw 1	60	High (180)
1st air intake purge	10	High (180)
2nd sample gas purge	180	High (180)
**Data Analysis Configuration Settings**		
Algorithm:	Canonical Discriminant Analysis (CDA)	
Preprocessing:	Auto-scaling	
Normalization:	Normalization 1	
Identification quality:	Always choose	

**Table 3:** Cyranose 320^®^ eNose instrument settings for analysis of nasal swab samples as displayed in the PCnose™ software provided with the device.

**Class Assignment**	**Class Name**	**Training Samples**
Class 1	Pre-Challenge	5
Class 2	Post-Challenge	5
**Flow Operations**	**Time (seconds)**	**Pump Speed (cm^3^/minute)**
Baseline purge	10	Medium (120)
Sample draw 1	30	Medium (120)
1st air intake purge	10	High (180)
2nd sample gas purge	90	High (180)
**Data Analysis Configuration Settings**		
Algorithm:	Canonical Discriminant Analysis (CDA)	
Preprocessing:	Auto-scaling	
Normalization:	Normalization 1	
Identification quality:	Always choose	

**Table 4:** Individual calf lung consolidation score at necropsy.

Animal ID	Total Lung Consolidation
1	11.61%
2	2.39%
3	12.09%
4 ^1^	22.50%
5	9.18%
6 ^1^	24.65%
7 ^1^	17.85%
8	11.10%
9	8.85%
10 ^1^	12.30%
11 ^1^	14.79%
12	10.72%

^1^ Day-13 respiratory samples collected from these animals were used to create the “Post-Challenge” training set class for expired air and nasal swab on the eNose.

**Table 5:** Correctly identified expired air and nasal swab samples by the Cyranose^®^ 320 eNose pre- and post-induced BRD challenge.

Sample Type	Pre-Challenge (Day 1–3) No. Correct/No. Tested (%)	Post-Challenge (Day 6–13) No. Correct/No. Tested (%)
Expired Air	30/31 (96.8%)	66/91 (72.5%)
Nasal Swab	29/31 (93.5%)	89/91 (97.8%)

## Data Availability

The original contributions presented in this study are included in the article. Further inquiries can be directed to the corresponding author.

## Abbreviations

The following abbreviations are used in this manuscript:

BRD	Bovine respiratory disease
CALA	Computer-aided lung auscultation
eNose	Electronic nose
VOCs	Volatile organic compounds
LAMP	Loop-mediated isothermal amplification
BHV-1	Bovine herpes virus-1
BT	Bovine nasal turbinate
CPE	Cytopathic effect
TCID50	50% Tissue culture infectious dose
BHI	Brain–heart infusion
PBS	Phosphate-buffered saline
ID	Internal diameter
CSV	Comma-separated values
CDA	Canonical Discriminant Analysis algorithm
CDAnalysis™	Chemometric data analysis software
P	Probability
SE	Standard error
